# Is tranexamic acid effective for all traumatic brain injury patients? a severity based systematic review and meta-analysis

**DOI:** 10.3389/fphar.2025.1677936

**Published:** 2025-12-09

**Authors:** Wentao Bian, Mengxia Qi, Lu Ding, Yixuan Lu, Kangling Yan, Ziqi Wang, Jiancheng Zhang, Ping Zhou

**Affiliations:** 1 Shaanxi Provincial People’s Hospital, Xi’an, Shaanxi, China; 2 School of Nursing, Zhejiang Chinese Medical University, Hangzhou, Zhejiang, China; 3 Department of Laboratory Medicine, Third Affiliated Hospital of Sun Yat-sen University, Guangzhou, China; 4 College of Life Sciences, Northwestern University, Xi’an, Shaanxi, China; 5 University of Sydney Camperdown, Sydney, NSW, Australia; 6 School of Health Management, Guangzhou Medical University, Guangzhou, China; 7 Department of emergency medicine, Sichuan Provincial People′s Hospital, University of Electronic Science and Technology of China, Chengdu, China

**Keywords:** tranexamic acid, acute brain injury, mortality, systematic review, meta-analysis

## Abstract

**Background:**

The effectiveness of tranexamic acid (TXA) in patients with traumatic brain injury (TBI) remains controversial and appears to vary with the severity of the injury. This systematic review and meta-analysis aimed to assess the impact of TXA on mortality in patients with mild to moderate TBI and severe TBI.

**Methods:**

A systematic search was conducted across PubMed, Embase, Web of Science, Cochrane Library database, Chinese CNKI database, and clinical trial repositories was conducted up to 1 May 2024. Studies comparing TXA with placebo were performed for relevant studies comparing TXA for mild to moderate and severe TBI were included. After literature screening, data were independently extracted and pooled using random-effects or fixed-effects models according to the magnitude of heterogeneity. Certainty of findings was assessed using the GRADE methodology.

**Results:**

Sixteen studies involving 15,015 patients were analyzed. TXA could significantly reduce the 28-day mortality in patients with mild to moderate TBI (RR, 0.71; 95% CI 0.60–0.85; I^2^ = 0%), supported by randomized controlled trials (RR: (0.74; 95% CI:0.62–0.89; I^2^ = 0%; high certainty) and cohort studies: (RR:0.47; 95% 0.26–0.86; I^2^ = 0%; low certainty). However, no mortality benefit was observed in severe TBI patients (RR, 1.05; 95% CI, 0.93–1.19; I^2^ = 21%), as demonstrated in RCTs (RR:0.98; 95% CI, 0.91–1.05; I^2^ = 0%; moderate certainty) and cohort studies (RR,1.23; 95% CI:1.08–1.4; I^2^ = 0%; low certainty).

**Conclusion:**

The findings suggest that the therapeutic effectiveness of TXA varies by the severity of brain injury. Post-injury administration of TXA significantly reduced 28-day mortality in patients with mild to moderate TBI (GCS: 9–15) but showed no benefit in patients with severe TBI (GCS: 3–8). Further research is needed to investigate the effect of TXA on thromboembolic events and to determine optimal dosing strategies, particularly for severe TBI patients.

## 1 Introduction

Traumatic brain injury (TBI) is a leading cause of mortality and morbidity worldwide (Liesemer et al., 2014; Maas et al., 2017). The yearly incidence of TBI is estimated at 50 million cases worldwide, with complicated intracranial hemorrhage being the most fatal complication (Taylor et al., 2017; Final results of MRC CRASH, 2005). According to the Glasgow Coma Scale (GCS), TBI is categorized into 3 severity levels based on the GCS: mild (GCS score 13–15), moderate (GCS score 9–12), and severe (GCS score 3–8) (Fakharian et al., 2018; Rundhaug et al., 2015).

Tranexamic acid (TXA) is an antifibrinolytic agent that reduces bleeding by inhibiting plasmin production and preventing fibrin degradation (Wang and Santiago, 2022). TXA is currently widely used in trauma patients (Ramirez et al., 2017), but its role in patients with traumatic brain injury remains controversial. The CRASH-3 (CRASH-3 trial collaborators, 2019) study demonstrated that TXA can reduce mortality in TBI patients; however, the benefits appears to vary among patients with different severity levels.

Given these findings, we hypothesized that TXA has different effects on patients with varying severities of traumatic brain injury. This study summarizes the efficacy of TXA in reducing mortality across different levels of TBI severity.

## 2 Methods

### 2.1 Search strategy

The meta-analysis was performed according to the Preferred Reporting Items for Systematic Reviews and Meta-Analyses (PRISMA) guidelines and adhered to the Guidelines for Meta-Analysis of Epidemiological Research, as outlined in the study protocol (Page et al., 2021) (ID: CRD42023422701). A comprehensive systematic literature search was conducted across multiple databases, including PubMed, Embase, Cochrane Libraries, Chinese CNKI Database, Web of Science, and clinical trial repositories, covering studies published up to 1 May 2024.

**FIGURE 1:**
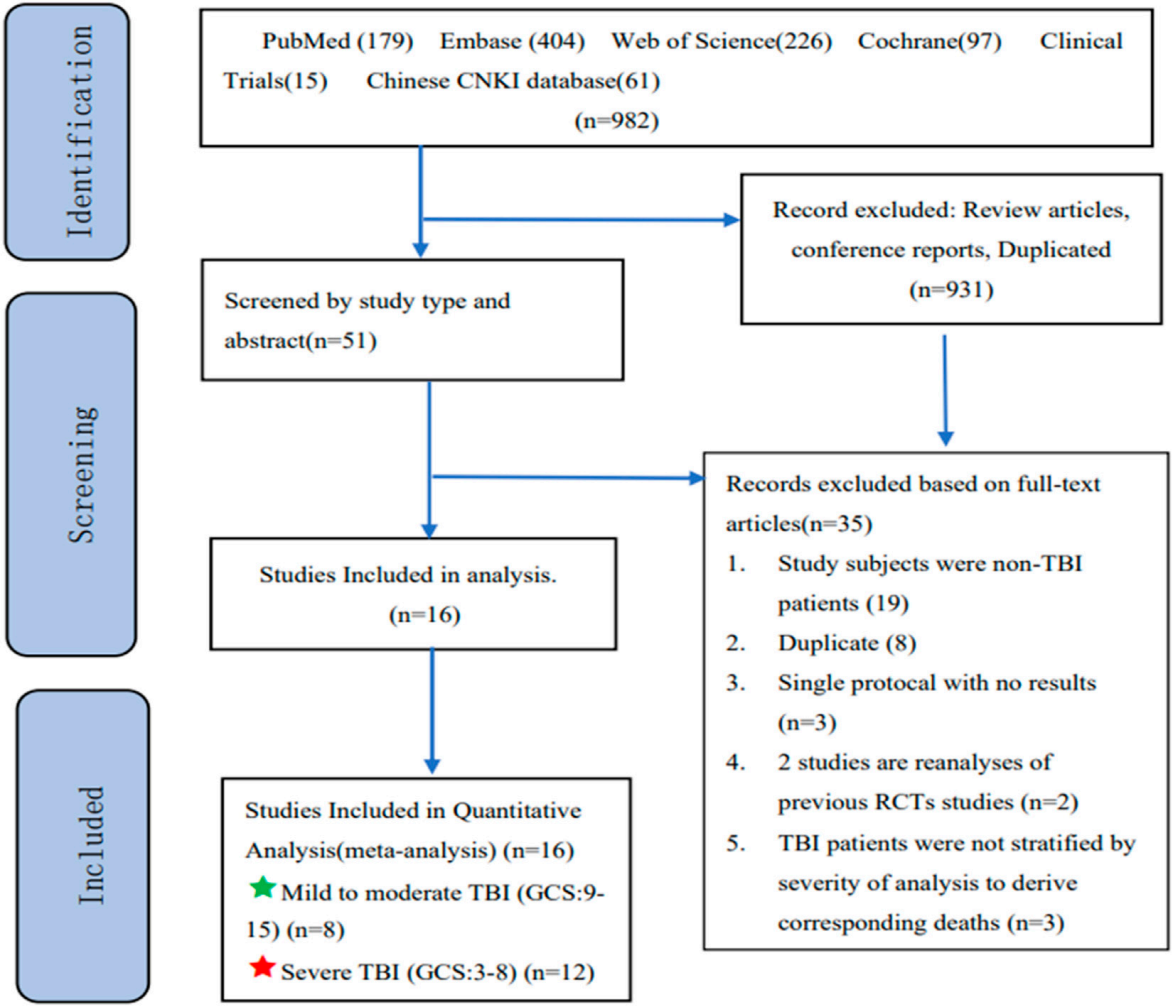
Flow diagram of the search strategy and study selection.

The search strategy utilized a combination of Medical Subject Headings (MeSH)/Emtree terms and title/abstract keywords, specifically “tranexamic acid” and “traumatic brain injury”. Detailed search strategies are provided in the Supplementary Material, with an overview of the process illustrated in Figure 1.

Two junior investigators independently screened the titles and abstracts of all identified studies to exclude those irrelevant to the research question. Subsequently, full texts of the screening articles were reviewed based on predefined screening criteria. The reference lists of included studies were also examined to identify additional relevant publications. Discrepancies in study selection or data extraction were resolved through discussions with two senior researchers.

### 2.2 Inclusion and exclusion criteria

The inclusion criteria for this study were as follows: (1) Eligible study types were randomized controlled trials (RCTs), cohort studies, or case-control studies; (2) Participants included patients aged 15 or older with acute TBI, confirmed by GCS score or CT scan showing any intracranial hemorrhage and no major extracranial hemorrhage; (3) There was a clear classification of the severity of brain injury, such as according to severity, Mild: GCS Score (13–15), Moderate: GCS Score (9–12), Severe: GCS Score (3–8); (4) The intervention is TXA treatment with intravenous infusion in any dosing regimen, and the control group is treated with placebo; (5) Reported deaths in patients with brain injury of varying severity, including 28-day, 30-day or hospital discharge deaths; (6) Reported related thromboembolic events, such as deep vein thrombosis or pulmonary embolism. The exclusion criteria are as follows: (1) Studies that did not involve patients with TBI; (2) Studies lacking a clear classification of brain injury severity (mild, moderate, and severe).

The PICOS (Population, Intervention, Comparator, Outcome, Study Design) criteria for inclusion and exclusion of studies into qualitative and quantitative meta-analysis are detailed in Supplementary Table S1.

### 2.3 Data extraction

Two junior researchers independently extracted data using predetermined forms. Any disagreements during the extraction process were resolved through discussion. Based on the characteristics of the included studies, patients with mild and moderate TBI were grouped into a single category for analysis. The data extracted included the following information: author and year of publication, country or region where study was conducted, patient demographics, including age (TXA/placebo groups) and percentage of males (TXA/placebo), number of patinets with mild to moderate TBI and severe TBI in the TXA group, mortality outcomes specifically the number of 28-day or hospital discharged deaths in mild to moderate and severe TBI patients (TXA), study design, TXA dosing and the timing of TXA administration after injury, as well as the reported primary outcomes.

### 2.4 Assessment of study quality

The 16 articles included in the analysis comprised RCTs and observational studies. The quality of these articles was evaluated using appropriate tools. For RCTs, we used the Cochrane Collaboration’s Risk of Bias tool (RoB 2) (Sterne et al., 2019) was used, which assess bias in six areas: (1) the randomization process, (2) deviations from intended interventions, (3) missing outcome data, (4) measurement of outcomes, (5) selection of reported results, and (6) overall bias.

For observational studies, the Risk of Bias in Non-randomized intervention Studies (ROBINS-I) (Sterne et al., 2016) was applied, evaluating bias in seven areas: (1) bias due to confounding, (2) participant selection, (3) classification of interventions, (4) deviations from intended interventions, (5) missing data, (6) measurement of outcomes, and (7) selection of reported results.

Additionally, certainty of the evidence for each outcome was assessed using the Grading of Recommendations, Assessment, Development, and Evaluation (GRADE) approach (Guyatt et al., 2008). Discrepancies in RoB or GRADE assessment were resolved through consensus among researchers. The Guideline Development Tool (https://www.gradepro.org) was used to create the Summary of Findings table.

### 2.5 Data analysis

Data analysis was conducted using R 4.3.1 software to generate correlation forest plots using both random effects models and fixed-effects models on the data. Discrepancies in the analysis were resolved through discussion. To assess the heterogeneity between trials, the chi-square test of homogeneity (where p < 0.1 indicates significant heterogeneity), and I^2^ statistics (values ≥50% considered reference values for potentially important heterogeneity) were used to assess heterogeneity between trials (Higgins et al., 2003). Subgroup analyses were performed based on study type, and the results of both random-effects and fixed-effects models were presented in forest plots. For conservative estimation, we believe that when I^2^ ≠ 0 we explain the pooled results of the random effects model; conversely, when I^2^ = 0, we explain the pooled results of the fixed effects model. In addition, we performed sensitivity analyses based on sample size and dose of TXA, with special attention given to trials with large sample sizes and trials with non-standard doses of TXA. Finally, we plotted colored funnel plots with outlines added to show relevant biases more clearly.

## 3 Results

### 3.1 Search result

Following the complete searching strategy, we identified a total of 982 records in the electronic database. After removing duplicates, screening titles, abstracts, and full texts where applicable, we included 10 reports of RCTs and 6 cohort studies for detailed evaluation (Figure 1). Due to the stratification of patients based on TBI in some trials, we identified 8 studies focusing on patients with mild to moderate TBI and 12 studies on patients with severe TBI.

### 3.2 Characteristics of included studies

The characteristics of the included studies are summarized in Table 1. A total of 10 RCTs and 6 cohort studies were included in the analysis. These studies collectively involved 15,015 patients with acute TBI. Of these, 7,245 patients had mild and moderate TBI, and 7,770 patients had severe TBI.

**TABLE 1:** Baseline characteristics of the studies included in the meta-analysis.

Study (author, year)	Region	Age	Male (%)	NO/NO death. Of mild to moderate (TXA)	NO./NO. death of severe (TXA)	Study design	TXA dose	Time of TXA administration after injury	Primary outcome
		TXA/placebo	TXA/placebo						
Hobensack and Phan. (2021)	US	41/42	73.2/74.8	17/352	19/90	Multisite RCT	A) TXA bolus 1 g, 10 min + Placebo bolus 10 min + Placebo infusion 8 h (1 g TXA) B) TXA bolus 1 g, 10 min + Placebo bolus 10 min + TXA infusion 8 h (2 g TXA) C) TXA bolus 1 g, 10 min + TXA bolus 10 min + TXA infusion 8 h (3 g TXA)	Prehospital	30-day mortality
CRASH-3 trial collaborators. (2019)	29 countries	41.7/41.9	80/80	166/2,846	689/1739	Multisite RCT	TXA bolus 1 g, 30 min, maintenance 1 g, 8h	<3 h	28days/hospital discharge death
Yutthakasemsunt et al. (2013)	Thailand	34.8/34.1	86/91	7/24	9/20	Single site RCT	1 g TXA bolus followed by 1 g TXA maintenance	NA	Death at hospital discharge
Bossers et al. (2021)	Netherlands	47/45	70/70	No	241/693	Prospective	TXA bolus 1 g	Prehospital	Death at 30 days
Rowell et al. (2020)	12 regions	39.5/36	73/75	No	93/657	Multisite RCT	A) 1 g bolus TXA and 1g maintenance TXA, 8 h B) 2 g bolus TXA, Placebo infusion	within 2 h	28-day mortality
Fakharian et al. (2018)	Iran	42.3/39.3	90.5/88	2/74	No	Single site RCT	The first dose was 1 g, followed by a maintenance dose of 1 g/1,000 mL for 8 h	within 8 h	deaths at hospital discharge
Chen. (2018)	China	38.2/40.9	65.4/42.3	1/26	No	Single site RCT	1 g, then 1 g after 8 h	NA	hospital mortality
Mousavinejad et al. (2020)	Iran	54.9/55.2	70/60	No	3/20	Single site RCT	1 g TXA bolus followed by 1 g TXA maintenance	within 8 h	28-day mortality
Ebrahimi et al. (2019)	Iran	40.2/40.6	85/85	No	2/20	Single site RCT	1 g TXA bolus followed by 1 g TXA maintenance	within 8 h	hospital discharge
de Tymowski et al. (2017)	US	39/36	73/75	No	53/312	Multisite RCT	1 g, then 1 g after 8 h	prehospital	28-day mortality
Chakroun-Walha et al. (2019)	Tunisia	44/39		No	27/96	Single site RCT	1 g, then 1 g after 8 h	as soon as possible	28-day mortality
Morte et al. (2019)	US	24.7/25.3	100/100	0/28	0/18	Prospective	Inject 1 g of TXA, followed by an infusion of 1 g over the next 8 h	within 3 h	hospital mortality
Van Wessem et al. (2022)	Netherlands	42/53	67/68	No	32/120	Prospective	TXA dosage was 1 g bolus; 1 g infusion was repeated over 8 h	within 3 h	30-day mortality
Valle et al. (2014)	US	42/43	85/86	No	13/32	Retrospective	1 g, then 1 g after 8 h	within 3 h	hospital mortality
Shiraishi et al. (2017)	Japan	56/57	72/74	25/188	No	Retrospective	TXA bolus 1 g, 30 min, maintenance 1 g, 8h	within 3 h	28-day mortality
Walker et al. (2020)	US	24.2/25.5	100/100	1/14	No	Retrospective	Tranexamic acid	NA	28-day mortality

In the included quantitative synthesis are 8 articles (5 RCTs and 3 cohort studies) focused on patients with mild to moderate TBI, and 12 articles (8 RCTs and 4 cohort studies) focused on patients with severe TBI. This divison reflects that some studies, such as CRASH-3, included patients across both mild/moderate and severe TBI categories.

Four studies used non-standard dosing regimens of TXA (CRASH-3 trial collaborators, 2019; Hobensack and Phan, 2021; Yutthakasemsunt et al., 2013; Morte et al., 2019). One study (Hobensack and Phan, 2021) administered TXA at doses of 1 g (151 patients), 2 g (141 patients), and 3 g (150 patients). Another study (Rowell et al., 2020) reported doses of 1 g (312 patients) and 2 g (345 patients). A third study (Bossers et al., 2021) administered a uniform dose of 1 g to all TBI patients, while a fourth study (Walker et al., 2020) did not specify the relevant dose of TXA. In contrast, the standard dosing regimen in the remaining studies involved a 1 g TXA bolus followed by 1 g TXA maintenance/Patients receiving non-standard doses represented 17.7% of the total study population. Three articles (Yutthakasemsunt et al., 2013; Walker et al., 2020; Chen, 2018) did not report the time window for TXA administration. For the remaining articles, reported administration of tranexamic acid was reported within 8 h of injury. In addition, we summarized the characteristics of the excluded studies, as detailed in Supplementary Table S2.

### 3.3 Quality assessment results

**FIGURE 2:**
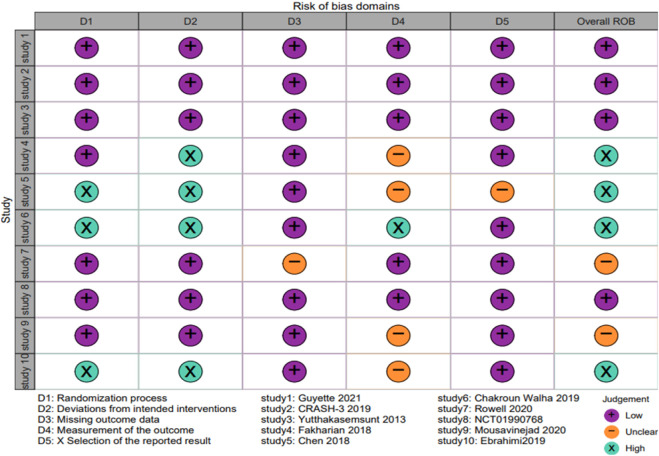
The risk of bias graph for randomized controlled trials based on ROB-2.

**FIGURE 3:**
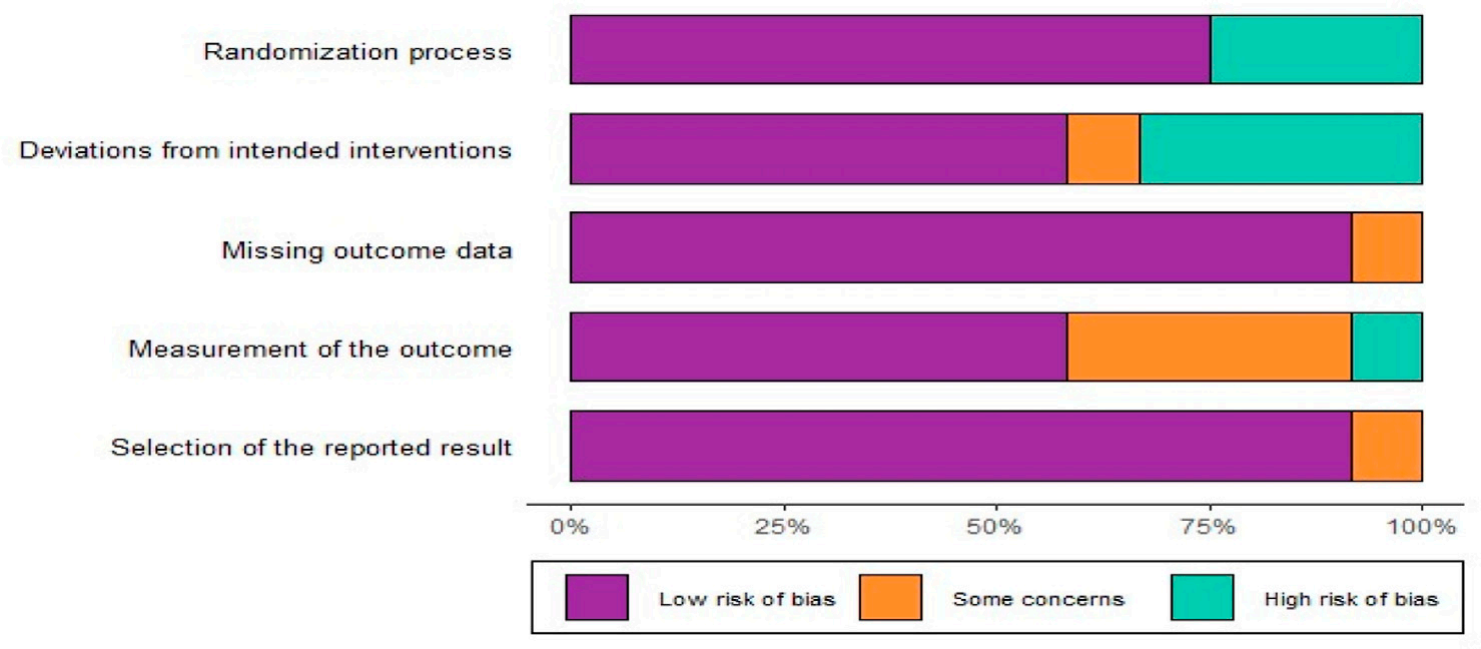
The risk of bias summary for randomized controlled trials based on ROB-2.

Among the 10 RCTs analyzed, four studies were identified as having a high risk of bias using the Cochrane RoB-2 tool, two trials were categorized as high RoB, and four trials were assessed as having a low RoB (Figures 2, 3). Due to the unique nature of the studies, allocation concealment was often challenging to achieve. Nevertheless, all included RCTs provided complete and non-selective outcome data. For the six cohort studies, two articles limited participant populations to war injuries, posing a significant bias risk based on the ROBINS-I tool (Supplementary Figures S1,S2). These limitations underline the variability in study design and participant selection across the included studies.

### 3.4 Outcomes

**FIGURE 4:**
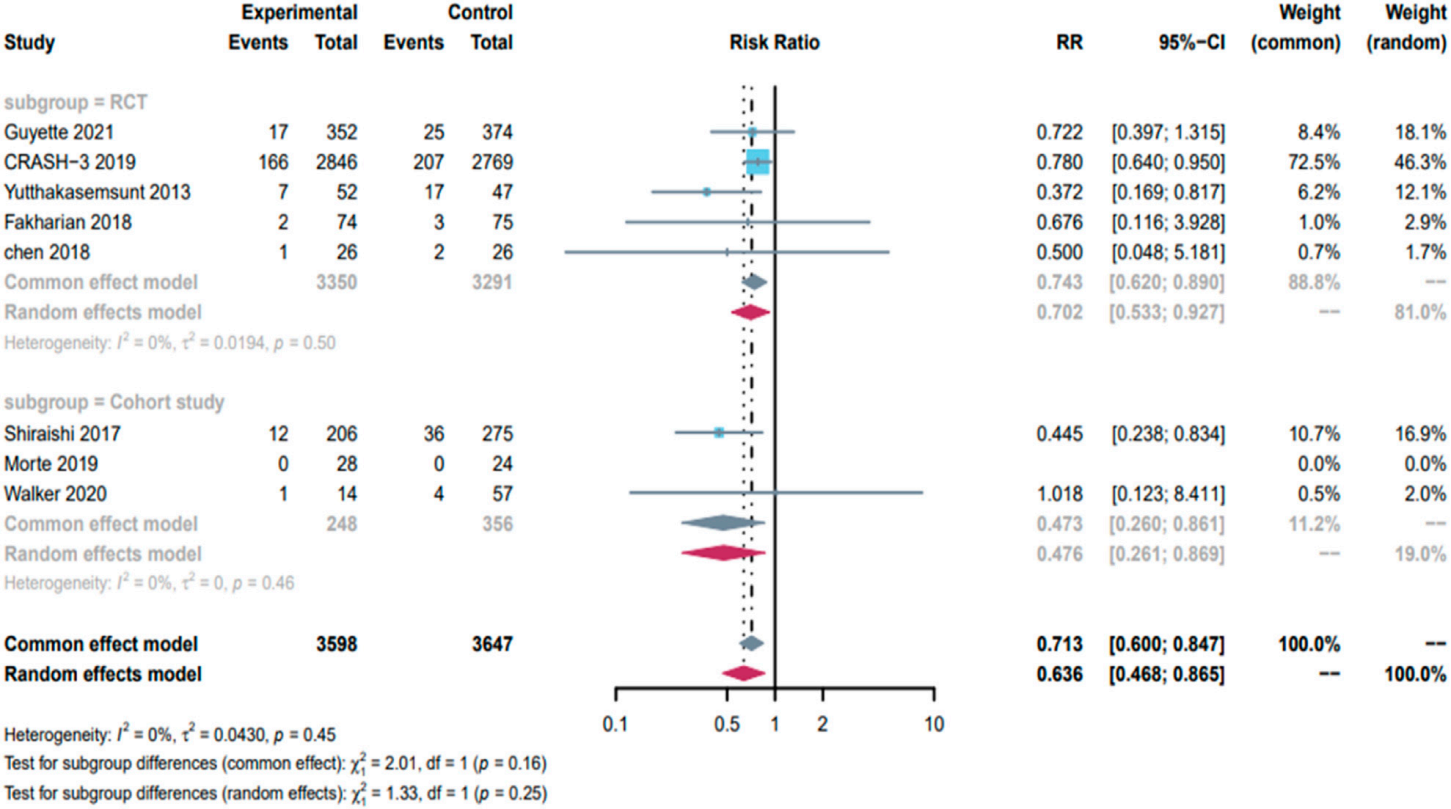
Forest plot of 28-day mortality in patients with mild to moderate TBI(GCS:9–15).

Figure 4. Shows the effect of TXA on 28-day mortality in patients with mild to moderate TBI, based on data from 5 RCTs and 3 cohort studies, comprising 3,598 patients in the TXA group and 3,647 in the control group. The results showed that TXA can significantly reduce 28-day mortality in patients with mild to moderate TBI (RR = 0.71; 95% CI, 0.60–0.85; P = 0.45; I^2^ = 0%). Subgroup analyses were performed according to study type showed that TXA was effective in reducing mortality in both the RCTs subgroup (RR = 0.74; 95% CI, 0.62–0.89; I^2^ = 0%) and the cohort study subgroup (RR = 0.47; 95% CI, 0.26–0.86; I^2^ = 0%) were shown to reduce 28-day mortality in patients with mild to moderate TBI.

**FIGURE 5:**
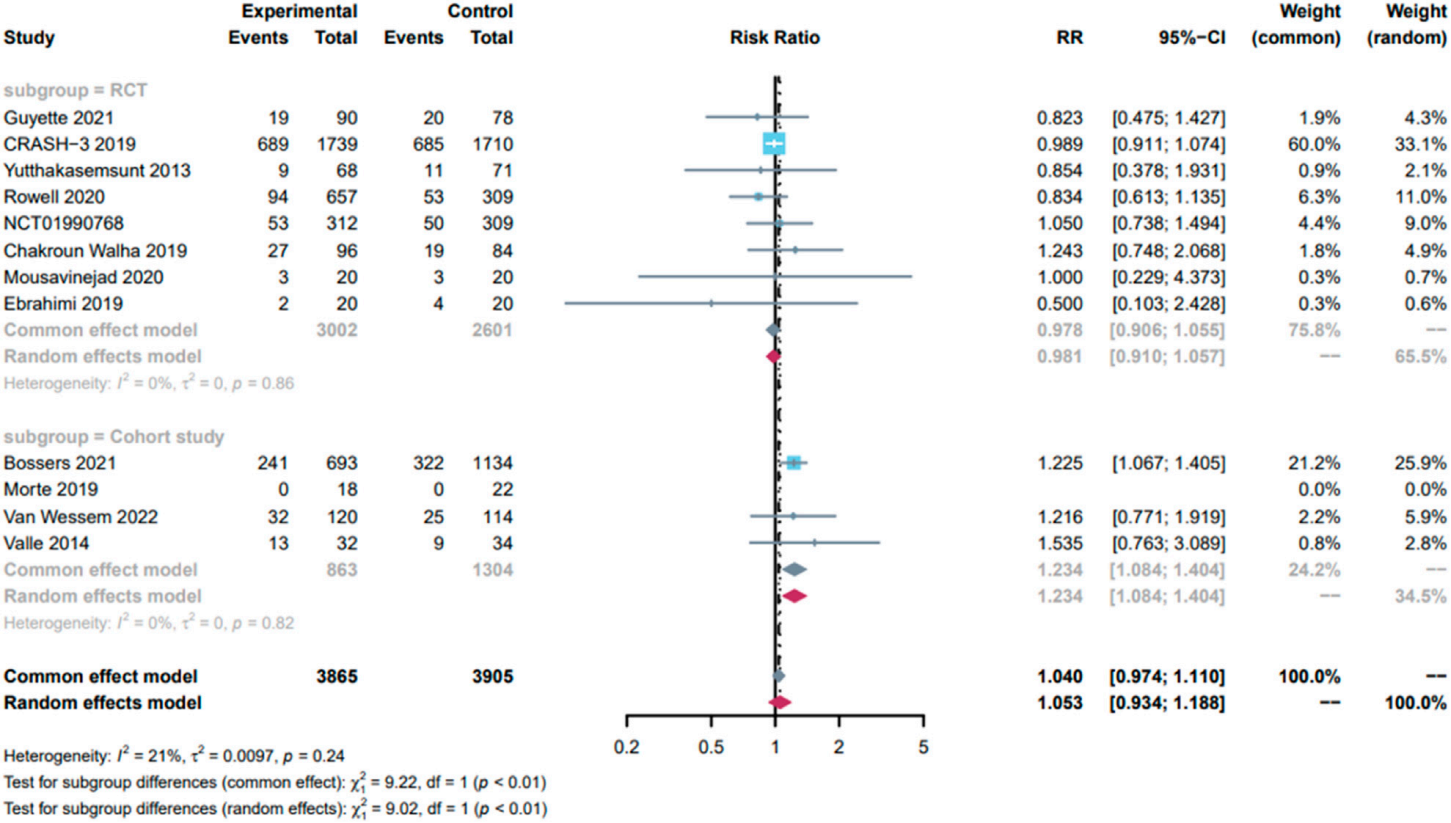
Forest plot of 28-day mortality in patients with severe TBI(GCS:3–8).

Figure 5 presents the effect of TXA on 28-day mortality in patients with severe TBI, based on 5 RCTs and 3 cohort studies with 3,865 in the TXA group and 3,905 in the control group. The findings indicated that TXA did not reduce 28-day mortality in patients with severe TBI (RR = 1.05; 95% CI, 0.93–1.19; P = 0.24; I^2^ = 21%). Subgroup analyses were performed according to study type. TXA did not reduce 28-day mortality in patients with severe TBI in the RCTs subgroup (RR = 0.98; 95% CI, 0.91–1.06; I^2^ = 0%) but increased 28-day mortality in patients with severe TBI in a cohort study subgroup (RR = 1.23; 95% CI, 1.08–1.40; I^2^ = 0%).

Table 2 summarizes the findings for all outcomes including the certainty of evidence. A comprehensive analysis of RCTs found that TXA reduced 28-day mortality in patients with mild-to-moderate TBI [RR 0.74; 95% CI 0.62–0.89; risk difference (RD) 1.0% reduction; 95% CI 0.8% reduction to 2.9% increase; high degree of certainty], but not in patients with severe TBI [RR 1.05; 95% CI 0.93–1.19; risk difference (RD) reduction 0.6%; 95% CI-1.6%–2.9%; moderate certainty], cohort studies, thromboembolic complications, and analysis of patients with a GOS score less than 4 were determined to have low or very low certainty.

**TABLE 2:** GRADE summary of findings.

Certainty assessment							No of patients		Effect		Certainty	Importance
No of studies	Study design	Risk of bias	Inconsistency	Indirectness	Imprecision	Other considerations	TXA	Placebo	Relative (95% CI)	Absolute (95% CI)		
Mortality in patients with mild to moderate TBI(GCS:9–15)												
5	RCT	Not serious	Not serious	Not serious	Not serious	None	193/3,350 (5.8%)	254/3,291 (7.7%)	RR 0.74 (0.62–0.89)	10 fewer per 1,000 (from 8 fewer to 29 more)	⊗⊗⊗⊗ HIGH	CRITICAL
Mortality in patients with mild to moderate TBI(GCS:9–15)												
3	Cohort studies	Serious^a^	Not serious	Not serious	Serious^b^ ^,^ ^c^	None	13/248 (5.2%)	40/356 (11.2)	RR 0.47 (0.26–0.86)	60 fewer per 1,000 (from 16 fewer to 83 more)	○⊗⊗○ LOW	CRITICAL
Mortality in patients with severe TBI(GCS:3–8)												
8	RCT	Not serious	Not serious	Not serious	Serious^b^	None	896/3,002 (29.8%)	845/2,601 (32.5%)	RR 0.98 (0.91–1.05)	6 fewer per 1,000 (from 29 fewer to 16 more)	○○○⊗ MODERATE	CRITICAL
Mortality in patients with severe TBI(GCS:3–8)												
4	Cohort studies	Serious^d^	Not serious	Not serious	Serious^b^	None	286/863 (33.1%)	356/1,304 (27.3%)	RR 1.23 (1.08–1.4)	63 fewer per 1,000 (from 22 fewer to 109 more)	○⊗⊗○ LOW	CRITICAL
Thromboembolic complications (GCS:9–15)												
2	All	Serious^e^	Not serious	Serious^f^	Very serious^b^ ^,^ ^c^	None	3/276 (1.1%)	5/276 (1.8%)	RR 0.6 (0.14–2.48)	63 fewer per 1,000 (from 22 fewer to 109 more)	⊗○⊗⊗ VERY LOW	IMPORTANT
Thromboembolic complications (GCS:3–8)												
3	RCT	Not serious	Serious^g^	Serious^h^	Serious^b^ ^,^ ^c^	None	67/1,065 (6.3%)	70/702 (10.0%)	RR 0.72 (0.25–2.04)	28 fewer per 1,000 (from 75 fewer to 104 more)	⊗○○○ VERY LOW	IMPORTANT
Unfavorable outcome at discharge (GOS) (GCS:9–15)												
2	RCT	Not serious	Not serious	Not serious	Very serious^b^ ^,^ ^c^	None	11/100 (11%)	19/101 (18.8%)	RR 0.58 (0.29–1.16)	79 fewer per 1,000 (from 134 fewer to 30 more)	⊗⊗○○LOW	IMPORTANT
Unfavorable outcome within 6 months after discharge (GOS) (GCS:9–15)												
2	RCT	Not serious	Not serious	Not serious	Very serious^b^ ^,^ ^c^	None	6/100 (6%)	15/101 (14.9%)	RR 0.40 (0.16–1.00)	89 fewer per 1,000 (from 125 fewer to 0 more)	⊗⊗○○LOW	IMPORTANT
Unfavorable outcome at discharge (GOS) (GCS:3–8)												
2	ALL	Serious^i^	Serious^j^	Not serious	Serious^b^	None	930/1,412 (65.9%)	953/1,510 (63.1%)	RR 1.05 (0.93–1.19)	32 fewer per 1,000 (from 44 fewer to 120 more)	○○⊗○ VERY LOW	IMPORTANT

Explanations:

^a^ One studies (Walker 2020) may have serious bias, and their contribution weight to this outcome is 9.4%, which lower our certainty in effect.

^b^ Wide confidence intervals do not exclude important benefit or harm which lowers our certainty in effect.

^c^ Low number of events below optimal information size contributing to imprecision which lowers our certainty in effect.

^d^ Two studies (Van Wessem 2022; Valle 2014) may have serious bias, and their contribution weight to this outcome is 3.05%, and 10.8%, respectively, which lower our certainty in effect.

^e^ One studies (chen 2018) may have high ROB, and their contribution weight to this outcome is 9.4%, respectively, which lower our certainty in effect.

^f^ Chen 2018 do not mention how they identifed DVT/PE.

^g^ High I2 (85%) and non-overlapping confidence intervals indicate important inconsistencies.

^h^ Rowell 2020 and Chakroun Walha 2019 do not mention how they identifed DVT/PE.

^i^ One study (Chakroun Walha 2019) has high ROB, and one study (Rowell 2020) has probably high ROB, and their contribution weight to this outcome is 6.2% and 24.5% respectively, which lower our certainty in effect.

^j^ High I2 (67%) and non-overlapping confidence intervals indicate important inconsistencies.

### 3.5 Sensitivity analyses and publication bias

We used R 4.3.1 software to conduct sensitivity analysis and publication bias. Sensitivity analysis showed no significant differences between the pooled results for calculations that did not exceed the 95% confidence limit (Supplementary Figures S6,S7). We also generated funnel plots to assess publication bias (Supplementary Figures S8,S9. However, the funnel plot showed significant asymmetry between studies (Supplementary Figure S8), suggesting possible publication bias.

## 4 Discussion

This systematic review and meta-analysis show that TXA reduces 28-day mortality in patients with mild to moderate TBI (GCS: 9–15) but not in patients with severe TBI (GCS: 3–8). Due to the small sample size and limited number of events, the current low-certainty evidence may suggest that TXA does not significantly impact disability outcomes in TBI of varying severity, nor does it increase the risk of thromboembolic complications.

The findings of the CRASH-3 trial are particularly noteworthy. In fact, our results are also consistent with the relevant conclusions of the trial (CRASH-3 trial collaborators, 2019). Standard doses of TXA appear to have varying benefits depending on TBI severity. Because TXA can inhibit the fibrinolytic system, it can pass through the blood-brain barrier and reduce the level of fibrinolysis early after injury (Supplementary Figure S10), thereby reducing the expansion of intracranial hematoma, so it is suitable for central nervous system hemorrhage (Jokar et al, 2017; Maegele, 2021; CRASH-2 Collaborators Intracranial Bleeding Study, 2011). Previous studies have shown that TXA can significantly reduce the haematoma size in patients with cerebral hemorrhage (Safari et al., 2020), and the extent of hematoma is closely associated with patients’ prognosis (Zille et al., 2022). It is worth reminding that these TBI patients did not have severe damage to other organs. However, in cases of severe TBI, the large volume of intracranial hemorrhage often leads to symptoms such as brain herniation, and pupil insensitivity to light reflex may appear in the early stage of trauma, and the use of tranexamic acid may not be effective at this time (Maegele, 2021; Roberts et al., 2013). It is worth noting that the cohort study shows that tranexamic acid can increase the mortality of patients with severe TBI. Possible explanations for this evidence may be related to the hypercoagulable state after severe brain injury that may promote cerebral vascular microthrombosis or systemic diffuse intravascular coagulation (Harhangi et al., 2008). In addition, since patients with severe brain trauma have a considerable risk of deep venous thrombosis (For the EPO-TBI investigators and the ANZICS Clinical Trials Group et al., 2017), the use of TXA may break the balance mechanism of anticoagulation and fibrinolysis, which may lead to poor prognosis. In addition, as the severity of injury in patients with severe TBI increases, extracranial injury and unmeasured confounding factors may also lead to an increased risk of death in such patients. These findings highlight the need for future research to determine appropriate TXA dosing for patients with severe TBI.

Three meta-analyses on TXA for all-cause mortality in TBI patients have been previously published (Weng et al., 2019; July and Pranata, 2020; Lawati et al., 2021), but none performed stratified analyses based on the severity of TBI patients. July et al.'s study (July and Pranata, 2020) showed that TXA can reduce mortality in TBI patients but included non-TBI patients in the CRASH-2 trial in their analysis. To ensure a more robust conclusion, we included only TBI patients from the CRASH-2 trial in our analysis. The meta-analysis published by Weng et al. (Weng et al., 2019) reported an impact of TXA on all-cause mortality in TBI patients: RR: 0.64 (0.41–1.00), but they included three small sample trials previously published. In contrast, our meta-analysis conducted a detailed search and included all relevant studies, including the CRASH-3trial. The meta-analysis published by Lawati et al. (Lawati et al., 2021) showed that TXA could not reduce the mortality of patients with traumatic brain injury [ RR0.95 (0.88–1.02) ]. They failed to stratify the severity of TBI patients, as the wider confidence interval buried the benefits of some mild to moderate TBI, as shown in the stratification results of the CRASH-3 trial.

Due to the lack of robust data on the side effects of TXA in TBI patients of different severity levels, the limited sample size and number of events indicate that there is no statistical difference in thromboembolic events between the TXA group and the placebo group in TBI patients of different severity levels, which is consistent with the results of this large randomized controlled trial previously published (CRASH-3 trial collaborators, 2019; Hobensack and Phan, 2021; Rowell et al., 2020). However, the relatively low level of evidence for related complications, as assessed in GRADE, creates uncertainty about whether TXA use might increase the incidence of thromboembolic or other complications. Future research should explore the benefits of TXA in patients with mild, moderate, and severe TBI as well as its impact on specific types of hemorrhage, including subdural, subarachnoid, and cerebral parenchymal hemorrhage. Related thromboembolic events can also be explored in greater detail across different TBI severity levels. Notably, most of the studies included in this analysis administered TXA at a standard dose of a 1 g bolus followed by a 1 g maintenance dose within 3 h post-injury. However, this standard dose of TXA has proven ineffective for patients with severe TBI. Therefore, further research is essential to identify the optimal TXA dosing regimen for severe TBI patients to improve outcomes.

This study has several advantages. Firstly, it is the first study to stratify TBI patients by severity, highlighting the potential benefits of TXA for patients with mild to moderate TBI. Secondly, it includes a comprehensive literature search of all relevant studies and incorporates a GRADE evaluation to assess the certainty of evidence. However, there are also limitations. First of all, although we try to extract data exclusively from relevant trials involving isolated TBI patients, very few trials still include cases of mild extracranial injury. In addition, this study primarily focuses on the effects of standard-dose TXA on patients with varying TBI severity. While a few studies utilized non-standard dosing regimens, their proportions were relatively small, and we conducted a subgroup analysis to show that after excluding trials with non-standard doses of TXA, the results were consistent with the conclusions of this article (Supplementary Figures S3, S4, S5). Furthermore, the available evidence, particularly from heavily weighted studies at high risk of bias, is insufficient to establish a definitive causal relationship between TBI and the outcomes studied. Moreover, we see that these studies from different development regions show that, due to the economic disparity, patients with severe traumatic brain injury in less developed regions may have a higher mortality rate compared to those in more developed regions, depending on the effectiveness of treatment. Therefore, the associations presented in this meta-analysis must be interpreted with caution. Future studies with more rigorous designs, more transparent reporting, and better control for key confounders are needed to increase the certainty of the evidence.

## 5 Conclusion

In conclusion, the findings suggest that the therapeutic effectiveness of tranexamic acid varies with the severity of the traumatic brain injury. When administered early after injury, TXA significantly reduces 28-day mortality in patients with mild to moderate TBI (GCS:9–15) but does not demonstrate the same benefit in severe TBI patients (GCS:3–8). Further research is required to better understand the effect of TXA on thromboembolic events and to explore optimal dosing strategies, particularly for patients with severe TBI.
