# Supplementary material for: Is tranexamic acid effective for all traumatic brain injury patients? a severity based systematic review and meta-analysis
Source: Front Pharmacol. 2025 Dec 9;16:1677936. doi: 10.3389/fphar.2025.1677936 (PMC12722793; doi:10.3389/fphar.2025.1677936)
Supplement: Supplementary file 1 [file Supplementaryfile1.docx]

**Supplementary material**

**Supplemental Figures**

**ROBINS-I quality evaluation**

Figure S1. The risk of bias graph for cohort studies based on ROBINS-I…………. ….. pag. 2

Figure S2. The risk of bias summary for cohort studies based on ROBINS-I……. pag. 2

**Forest plots**

Figure S3. Forest plots excluding TXA administration doses of 1g or 3g {mild to moderate TBI(GCS:9-15)} ……………………………………………………….……pag. 3

Figure S4. Forest plot of 28-day or discharge mortality in patients with TBI at TXA doses of 1 g and 3 g. ……………………………………………………………………………pag. 3

Figure S5. Forest plot of 28-day or discharge mortality in patients with TBI at TXA doses of 1 g and 3 g. ……………………………………………………………..…………..…pag. 3

**Sensitivity Analysis**

Figure S6. Mortality in patients with mild to moderate TBI(GCS:9-15) ….……..…pag. 4

Figure S7. Mortality in patients with severe TBI(GCS:3-8) …………….…...………pag. 4

**Publication bias-funnel plot**

Figure S8. Funnel plot of 28-day mortality in patients with mild to moderate TBI...pag. 4

Figure S9. Funnel plot of 28-day mortality in patients with severe TBI………….. pag. 5

**Fig S10.** The mechanism of action of tranexamic acid………….…………...………pag. 5

**Supplemental tables**

**Table 1.** PICOS Criteria for Inclusion and Exclusion of Studies into Qualitative/Quantitative Meta-analysis……………………….………………………………………………..……pag. 6

**Table 2.** Characteristics of excluded studies……….…………………………………..…pag. 7

**Retrieval Strategy of Database**

PubMed search strategy….……………………………….……………….………..……pag. 10

Search strategy for Web of Science……………………. ….…………………….………pag. 11

Embase search strategy….……………………………….………………………………pag. 11

Cochrane search strategy…………………………….………………….………….……pag. 12

Search strategy for Clinical trials……………….……………….………………….……pag. 12

Search strategy for Chinese CNKI Database….………………….……….………..….…pag. 13

**Supplemental Figure**

**Fig S1.** The risk of bias graph for cohort studies based on ROBINS-I.


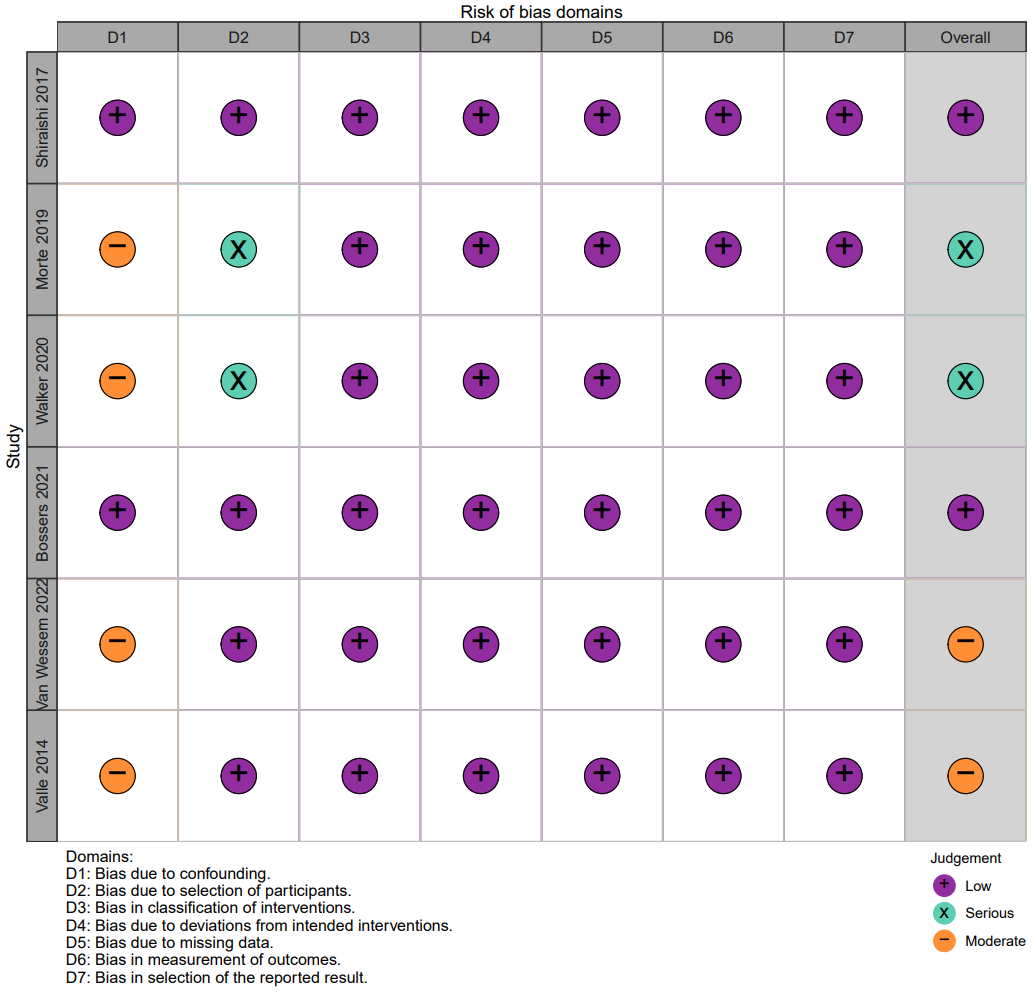


**Fig S2.** The risk of bias summary for cohort studies based on ROBINS-I


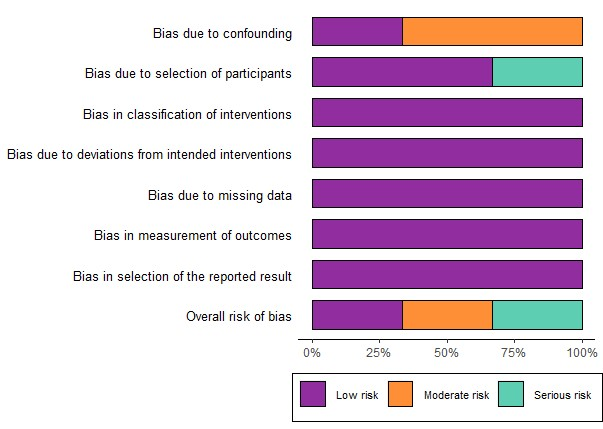


**Fig S3.** Forest plots excluding TXA administration doses of 1g or 3g {mild to moderate TBI(GCS:9-15)}


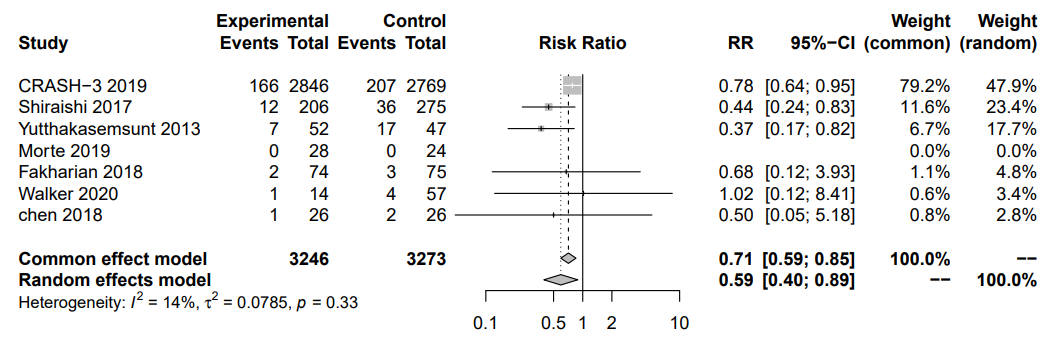


**Fig S4.** Forest plots excluding TXA administration doses of 1g or 3g {severe TBI(GCS:3-8)}


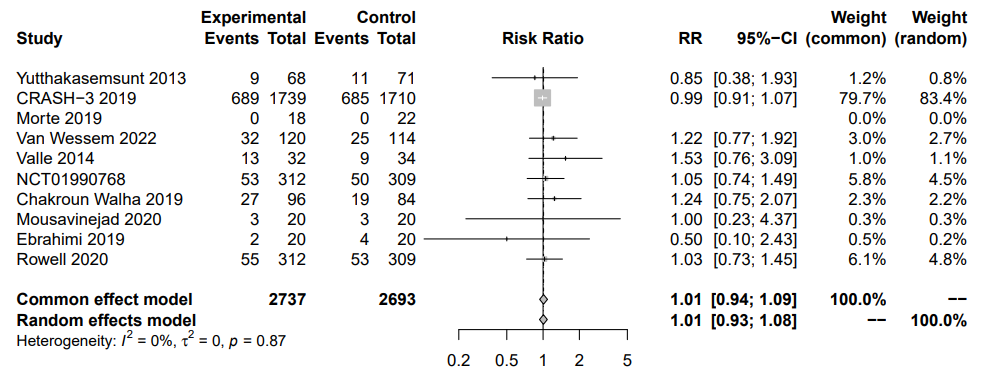


**Fig S5.** Forest plot of 28-day or discharge mortality in patients with TBI at TXA doses of 1 g and 3 g.


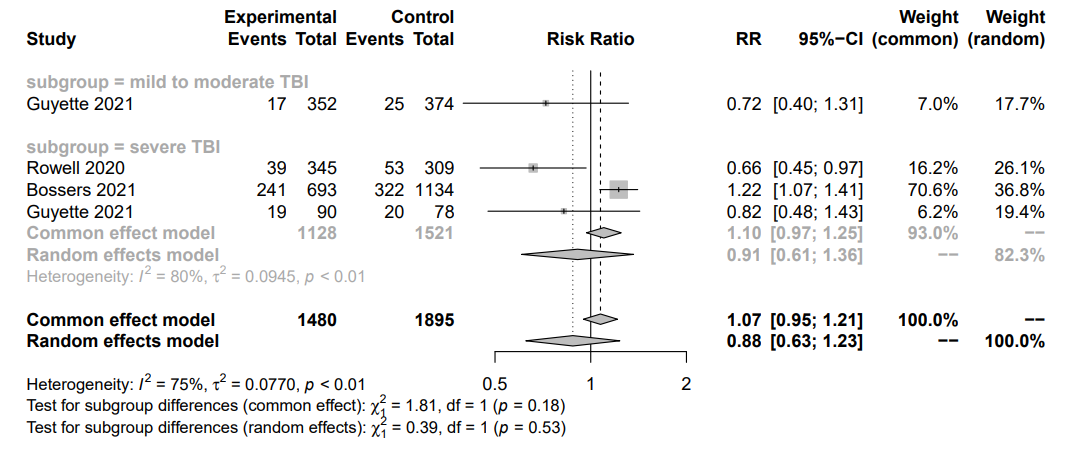


**Sensitivity Analysis**

**Fig S6.** Mortality in patients with mild to moderate TBI(GCS:9-15)

**
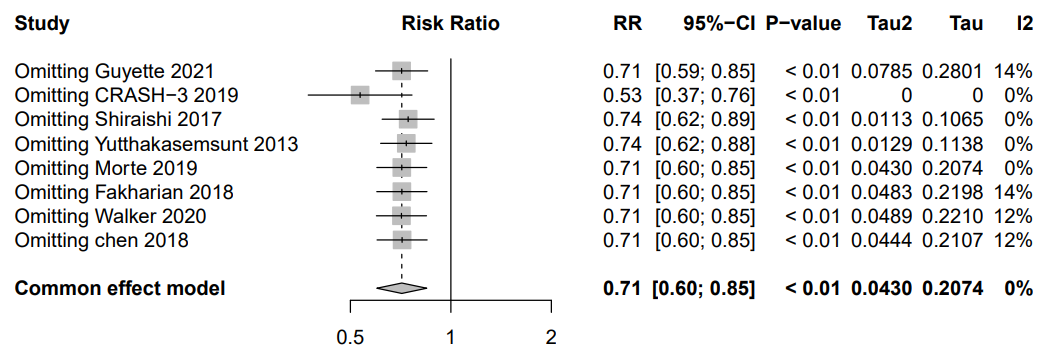
**

**Fig S7.** Mortality in patients with severe TBI(GCS:3-8)


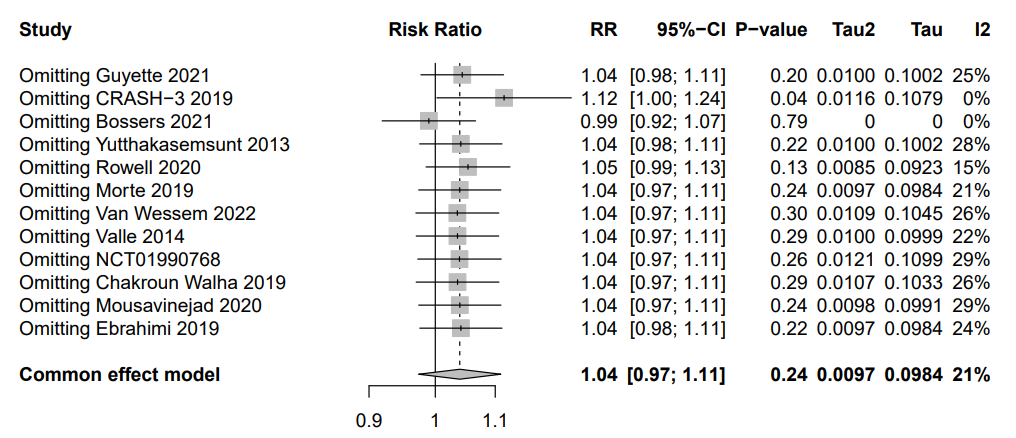


**Fig S8.** Funnel plot of 28-day mortality in patients with mild to moderate TBI. (RR, Relative Risk; s.e, standard error).


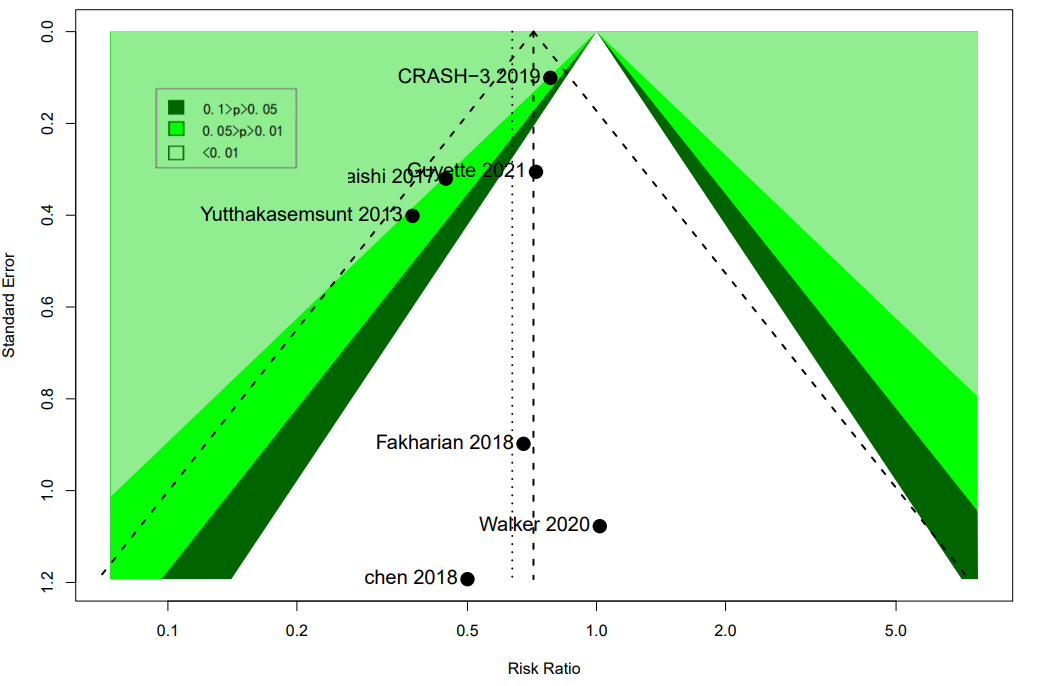


**Fig S9.** Funnel plot of 28-day mortality in patients with severe TBI (RR, Relative Risk; s.e, standard error


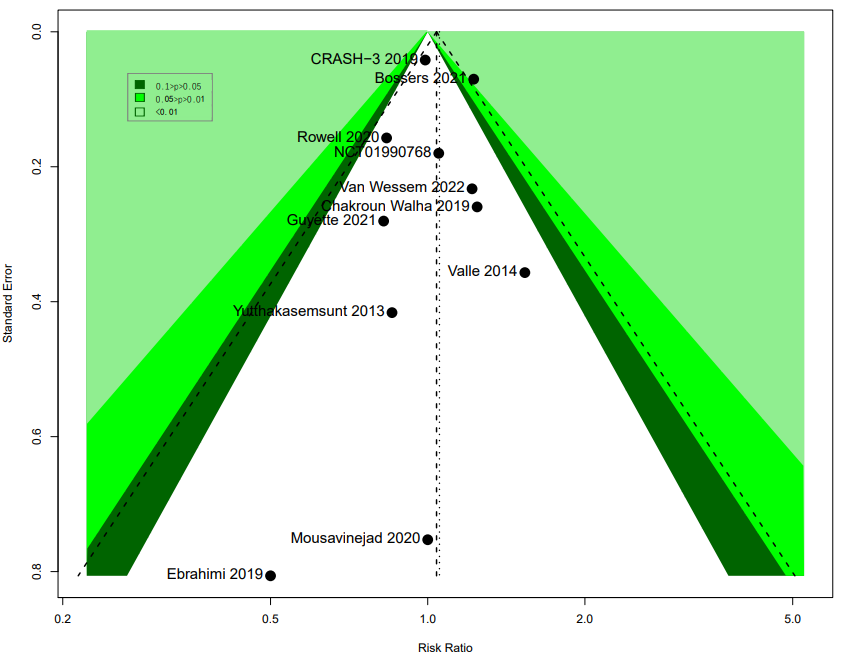


**Fig S10.** The mechanism of action of tranexamic acid


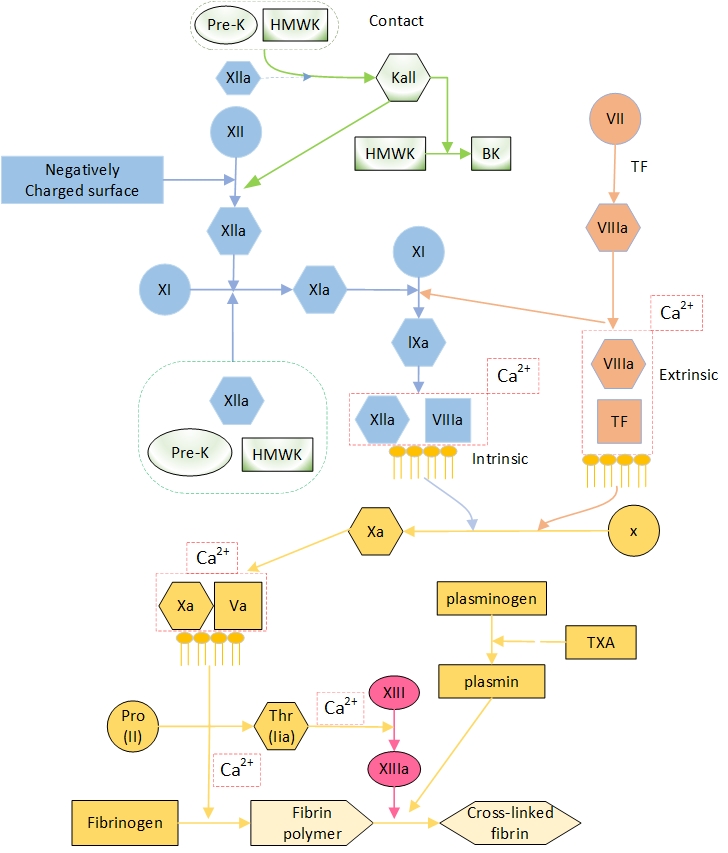


**Supplemental tables**

**Table 1. PICOS Criteria for Inclusion and Exclusion of Studies into Qualitative/Quantitative Meta-analysis**

| Parameter | Inclusion criteria | Exclusion criteria |
| --- | --- | --- |
| Patients | (1) Patients aged 15 or over with acute traumatic brain injury  (2) There was a clear classification of the severity of brain injury, such as according to severity, Mild: GCS Score (13-15), Moderate: GCS Score (9-12), Severe: GCS Score (3-8) | (1) Age ≤15 years  (2) Patients with non-traumatic brain injury or combined thoracoabdominal injuries  (3) Patients with traumatic brain injury of no clearly defined severity |
| Intervention | Treatment with any dose of intravenous tranexamic acid | Interventions were non-tranexamic acid therapy or a combination of tranexamic acid and other drugs |
| Comparator | Intravenous injection of the same dose of placebo | - |
| Outcomes | Primary: (1) **★**28-day or discharge mortality in patients with mild to moderate TBI(GCS:9-15)  (2) ★28-day or discharge mortality in patients with severe TBI(GCS:3-8).  Secondary:(1) Thromboembolic complications (GCS:9-15)  (2) Thromboembolic complications (GCS:3-8)  (3) Unfavorable outcome at discharge (GOS) (GCS:9-15)  (4) Unfavorable outcome within six months after discharge (GOS) (GCS:9-15)  (5) Unfavorable outcome at discharge (GOS) (GCS:3-8) | - |
| Study design | 1. Clinical randomized trials. 2. Prospective and retrospective cohort studies 3. case-control study | (1) Repeat publications of the same analysis or dataset  (2) Case reports  (3) Conference abstracts  (4) Opinion pieces  (5) Books or grey literature |

**Abbreviations:** PICOS, patients, intervention, comparator, outcomes, study design; GCS: Glasgow Coma Scale; GOS: Glasgow Outcome Scale.

**Table 2. Characteristics of excluded studies**

| Study (Author, Year) | Region | Study design | Number of Patients | Male (%) | | Inclusion criteria | TXA dose | Time of TXA administration after injury | Primary outcome | Comments |
| --- | --- | --- | --- | --- | --- | --- | --- | --- | --- | --- |
|  |  |  |  | **TXA** | **placebo** |  |  |  |  |  |
| Brito, 2023^(Supplemental referenec1)[1]^ | US | Multisite RCT | 649 | 73.5(2g) | 72.8(1g) | TBI Trial with blunt or penetrating injury and suspected TBI (GCS /=90) | A)  a 2-g TXA bolus  B) a 1-g bolus plus 1 g 8-hour infusion | within 2 hours of injury | 28-day mortality | The control group is not a placebo; The main comparison is the effectiveness of early administration of tranexamic acid in patients with TBI. |
| Mahmood, 2021^(Supplemental reference2)[2]^ | UK | Multisite RCT | 1767 | 79 | 81 | Adults with head injury who were within 3 hours of injury, and had a baseline Glasgow Coma Score (GCS) of ≤12or any intracranial bleeding on CT, and no significant extracranial bleeding were eligible for randomization in hospital | TXA bolus 1g, 30 min, maintenance 1g, 8h | < 3 h | The volume of IPH (ie, contusions) seen on the post-randomization scan | Patient population derived from re-analysis of CRASH-3 trial collaborators 2019 |
| Dixon, 2020^(Supplemental reference3)[3]^ | Canada and the United States | Multisite RCT | 700 | 75.3 | 79 | (1) patients 15 years or older, (2) blunt or penetrating TBI with an initial GCS score of 3 to 12, (3) systolic blood pressure of ≥90 mm Hg before randomization, and (4) at least one reactive pupil on initial assessment by EMS personnel | 1 g of TXA bolus followed by 1 g of TXA infusion for 8 hours | within 2 hours of injury | Comparison of TEG values (including LY30) between groups | The outcome measure is not mortality |
| Baidwan, 2023^(Supplemental reference4)[4]^ | US | a secondary cross-sectional analysis | 25,866 | 99.1 | 97.2 | a secondary cross-sectional analysis of previously published de-identified data from the DODTR which has been described elsewhere (included all patients in the DODTR aged 18 and above with a diagnosis of head injury) | TXA | NA | Mortality (at any time point) for patients who received prehospital or hospital TXA compared to those who did not receive TXA, specifically focusing on those with head injuries | Mortality rates that do not correspond to severity of traumatic brain injury |
| Gossiome, 2023^(Supplemental reference5)[5]^ | France | Multicenter, retrospective study | 934 | 70.2 | 67.7 | Mixed injuries including patients with isolated brain injury under 18 years of age | The median dosage was 16mg/kg | prehospital | TXA use in the prehospital setting (either at the scene or during  transportation) | Included patients were younger than 18 years old, with a mean age of 14 years |
| On behalf of the CRASH-3 trial collaborator, 2020^(Supplemental reference6)[6]^ | UK | Multisite RCT | 7637 | NA | NA | adults with TBI who were within 3 h of injury and had a Glasgow coma scale score (GCS) ≤ 12 or any intracranial bleeding on CT scan and no significant extra-cranial bleeding were eligible | TXA bolus 1g, 30 min, maintenance 1g, 8h | < 3 h | 228days/hospital discharge death | Patient population from CRASH-2 and CRASH-3 trials |
| CRASH-2 trial collaborators 2010, ^(Supplemental reference7)[7]^ | UK | Multisite RCT | 10096 | 83.6 | 84.0 | Adult trauma patients with significant hemorrhage (Systolic blood pressure <90 mm Hg or heart rate >110 beats per min, or both), or who were at risk of significant hemorrhage, and who were within 8 h of injury, were eligible for the trial. | Infuse a loading dose of 1 g of tranexamic acid over 10 minutes, followed by 1 g intravenously over 8 hours | within 8 hours of injury | 28days/hospital discharge death | Only some of the patients included in the CRASH-2 trial were patients with traumatic brain injury, accounting for 1.34%. |

**Abbreviations**: TXA: tranexamic acid

**Reference：**

1. Brito AMP, Schreiber MA, El Haddi J, et al (2023) The effects of timing of prehospital tranexamic acid on outcomes after traumatic brain injury: Subanalysis of a randomized controlled trial. J Trauma Acute Care Surg 94:86–92. https://doi.org/10.1097/TA.0000000000003767

2. Mahmood A, Needham K, Shakur-Still H, et al (2021) Effect of tranexamic acid on intracranial haemorrhage and infarction in patients with traumatic brain injury: a pre-planned substudy in a sample of CRASH-3 trial patients. Emerg Med J 38:270–278. https://doi.org/10.1136/emermed-2020-210424

3. Dixon AL, McCully BH, Rick EA, et al (2020) Tranexamic acid administration in the field does not affect admission thromboelastography after traumatic brain injury. J Trauma Acute Care Surg 89:900–907. https://doi.org/10.1097/TA.0000000000002932

4. Baidwan NK, Schauer LSG, Dixon JM, et al (2023) Tranexamic Acid Improves Survival in the Setting of Severe Head Injury in Combat Casualties

5. Gossiome A, Claustre C, Fraticelli L, et al (2022) Prehospital Tranexamic Acid in Major Pediatric Trauma Within a Physician-Led Emergency Medical Services System: A Multicenter Retrospective Study. Pediatric Critical Care Medicine 23:e507–e516. https://doi.org/10.1097/PCC.0000000000003038

6. On behalf of the CRASH-3 trial collaborators, Brenner A, Belli A, et al (2020) Understanding the neuroprotective effect of tranexamic acid: an exploratory analysis of the CRASH-3 randomised trial. Crit Care 24:560. https://doi.org/10.1186/s13054-020-03243-4

7. (2010) Effects of tranexamic acid on death, vascular occlusive events, and blood transfusion in trauma patients with significant haemorrhage (CRASH-2): a randomised, placebo-controlled trial. 376:

**Retrieval Strategy of Database**

**Pubmed：**

((((((((((((((AMCHA) OR (trans-4-(Aminomethyl)cyclohexanecarboxylic Acid)) OR (t-AMCHA)) OR (AMCA)) OR (Anvitoff)) OR (Cyklokapron)) OR (Ugurol)) OR (KABI 2161)) OR (Spotof)) OR (Transamin)) OR (Amchafibrin)) OR (Exacyl)) OR (Tranexamic acid)) AND ((((((((((((((((Brain Injury, Traumatic) OR (Traumatic Brain Injuries)) OR (Trauma, Brain)) OR (Brain Trauma)) OR (Brain Traumas)) OR (Traumas, Brain)) OR (TBI (Traumatic Brain Injury))) OR (Encephalopathy, Traumatic)) OR (Encephalopathies, Traumatic)) OR (Traumatic Encephalopathies)) OR (Injury, Brain, Traumatic)) OR (Traumatic Encephalopathy)) OR (TBIs (Traumatic Brain Injuries))) OR (TBI (Traumatic Brain Injuries))) OR (Traumatic Brain Injury)) OR (traumatic brain injury)))

**Web of science**

TS=((Tranexamic Acid OR AMCHA OR trans-4-(Aminomethyl)cyclohexanecarboxylic Acid OR t-AMCHA OR AMCA OR Anvitoff OR Cyklokapron OR Ugurol OR KABI 2161 OR Spotof OR Transamin OR Amchafibrin OR Exacyl)) AND TS=((Brain Injuries, Traumatic OR Brain Injury, Traumatic OR Traumatic Brain Injuries OR Trauma, Brain OR Brain Trauma OR Brain Traumas OR Traumas, Brain OR TBI (Traumatic Brain Injury) OR Encephalopathy, Traumatic OR Encephalopathies, Traumatic OR Traumatic Encephalopathies OR Injury, Brain, Traumatic OR Traumatic Encephalopathy OR TBIs (Traumatic Brain Injuries) OR TBI (Traumatic Brain Injuries) OR Traumatic Brain Injury))

**Embase**

Embase

Session Results

.......................................................

No. Query Results Results Date

#8. #6 AND #7 404 30 Apr 2023

#7. #3 OR #4 86,264 30 Apr 2023

#6. #1 OR #2 19,326 30 Apr 2023

2023

#4. 'brain injury, traumatic' OR 'traumatic brain 86,264 30 Apr 2023

injuries' OR 'trauma, brain' OR 'brain trauma' OR

'brain traumas' OR 'traumas, brain' OR 'tbi

(traumatic brain injury)' OR 'encephalopathy,

traumatic' OR 'encephalopathies, traumatic' OR

'traumatic encephalopathies' OR 'injury, brain,

traumatic' OR 'traumatic encephalopathy' OR 'tbis

(traumatic brain injuries)' OR 'tbi (traumatic

brain injuries)' OR 'traumatic brain injury'

#3. 'traumatic brain injury' 82,659 30 Apr 2023

#2. 'tranexamic acid' OR 'amcha' OR 19,317 30 Apr 2023

'trans-4-(aminomethyl)cyclohexanecarboxylic acid'

OR 't-amcha' OR 'amca' OR 'anvitoff' OR

'cyklokapron' OR 'ugurol' OR 'kabi 2161' OR

'spotof' OR 'transamin' OR 'amchafibrin' OR

'exacyl'

#1. tranexamic AND ('acid'/exp OR acid) 18,802 30 Apr 2023

**Cochrane：**

#1 Tranexamic acid 3746

#2 AMCHA OR t-AMCHA OR AMCA OR Anvitoff OR Cyklokapron OR Ugurol OR KABI 2161 OR Spotof OR Transamin OR Amchafibrin OR Exacyl OR Tranexamic acid 3774

#3 #1 and #2 3746

#4 traumatic brain injury 5033

#5 Brain Injury, Traumatic OR Traumatic Brain Injuries OR Trauma, Brain OR Brain Trauma OR Brain Traumas OR Traumas, Brain OR TBI Traumatic Brain Injury OR Encephalopathy, Traumatic OR Encephalopathies, Traumatic OR Traumatic Encephalopathies OR Injury, Brain, Traumatic OR Traumatic Encephalopathy OR TBIs Traumatic Brain Injuries OR TBI Traumatic Brain Injuries OR Traumatic Brain Injury 6578

#6 #4 and #5 5033

#7 #3 and #6 97

**Clinical trials:**

((((((((((((((AMCHA) OR (trans-4-(Aminomethyl)cyclohexanecarboxylic Acid)) OR (t-AMCHA)) OR (AMCA)) OR (Anvitoff)) OR (Cyklokapron)) OR (Ugurol)) OR (KABI 2161)) OR (Spotof)) OR (Transamin)) OR (Amchafibrin)) OR (Exacyl)) OR (Tranexamic acid)) AND ((((((((((((((((Brain Injury, Traumatic) OR (Traumatic Brain Injuries)) OR (Trauma, Brain)) OR (Brain Trauma)) OR (Brain Traumas)) OR (Traumas, Brain)) OR (TBI (Traumatic Brain Injury))) OR (Encephalopathy, Traumatic)) OR (Encephalopathies, Traumatic)) OR (Traumatic Encephalopathies)) OR (Injury, Brain, Traumatic)) OR (Traumatic Encephalopathy)) OR (TBIs (Traumatic Brain Injuries))) OR (TBI (Traumatic Brain Injuries))) OR (Traumatic Brain Injury)) OR (traumatic brain injury))) n=15

**Chinese CNKI Database:**

TKA=('氨甲环酸'+'氨甲基环己酸'+'抗血纤溶环酸'+'止血环酸'+'反式氨甲环酸+' Tranexamic Acid'+' Aminomethylcyclohexanoic acid '+' antifibrinolytic acid '+' Tournexic acid ')*(创伤性脑损伤 '+'脑损伤 '+'外伤性脑病'+'创伤性脑病'+' traumatic brain injury’+' Brain Trauma '+' Encephalopathy, Traumatic ') n=61
